# Target Localization via Integrated and Segregated Ranging Based on RSS and TOA Measurements

**DOI:** 10.3390/s19020230

**Published:** 2019-01-09

**Authors:** Slavisa Tomic, Marko Beko

**Affiliations:** 1 COPELABS, Universidade Lusófona de Humanidades e Tecnologias, Campo Grande 376, 1749-024 Lisboa, Portugal; beko.marko@ulusofona.pt; 2 ISR/IST, LARSyS, Universidade de Lisboa, Av. Rovisco Pais 1, 1049-001 Lisbon, Portugal; 3 CTS/UNINOVA, Campus da FCT/UNL, Monte de Caparica, 2829-516 Caparica, Portugal

**Keywords:** target localization, integrated measurements, received signal strength (RSS), time of arrival (TOA), critical distance

## Abstract

This work addresses the problem of target localization in adverse non-line-of-sight (NLOS) environments by using received signal strength (RSS) and time of arrival (TOA) measurements. It is inspired by a recently published work in which authors discuss about a *critical distance* below and above which employing combined RSS-TOA measurements is inferior to employing RSS-only and TOA-only measurements, respectively. Here, we revise state-of-the-art estimators for the considered target localization problem and study their performance against their counterparts that employ each individual measurement exclusively. It is shown that the hybrid approach is not the best one by default. Thus, we propose a simple heuristic approach to choose *the best* measurement for each link, and we show that it can enhance the performance of an estimator. The new approach implicitly relies on the concept of the critical distance, but does not assume certain link parameters as given. Our simulations corroborate with findings available in the literature for line-of-sight (LOS) to a certain extent, but they indicate that more work is required for NLOS environments. Moreover, they show that the heuristic approach works well, matching or even improving the performance of the best fixed choice in all considered scenarios.

## 1. Introduction

The aspiration for precise knowledge about the location of objects and/or people has motivated a great deal of scientific research recently [1,2,3,4,5,6,7,8,9,10,11,12,13,14,15,16,17,18,19,20,21,22]. This is due to a firm growth of the range of enabling devices and technologies, as well as the need for seamless solutions for location-based services. Besides localization precision, a common requirement for emerging solutions is that they are cost-restrained, both in terms of the financial and computational cost. Therefore, development of different localization strategies from already deployed technologies, e.g., from various terrestrial radio frequency sources is of great practical interest. Among others, these include concepts based on received signal strength (RSS), angle of arrival, time of arrival (TOA), or a combination of them [6,9,16,18,19,20,21,22].

Much work has been done regarding target localization based on integrated RSS and TOA measurements [18,19,20,21,22]. The authors in [23] and [20] studied the range estimation problem based on these two quantities. The authors in [23] employed the Newton-Raphson (NR) method to obtain a sub-optimal hybrid RSS-TOA ranging estimator for indoor non-line of sight NLOS environments in a closed-form. A joint ad-hoc (JAH) relaxation of hybrid likelihood function, which offers a good bias-variance trade-off to the derived ranging estimator was proposed in [20]. Although NR and JAH are originally designed for range estimation, after getting the range estimates, their generalization to the target localization problem is straightforward, for example through the use of a squared range approach [24]. In [18,21], the target localization problem in mixed line of sight (LOS)/NLOS environments was addressed. The authors in [18] first identified the type of the path for all links by using Nakagami distribution and then proposed a weighted least squares (WLS) estimator which utilizes TOA-only/RSS-only measurements if the link is identified as LOS/NLOS. An iterative squared range WLS estimator was proposed in [21]. The authors in [21] partially mitigate the negative influence of NLOS biases by approximating them by a single (mean) parameter, and then applying a simple alternating procedure to get an estimate of the target location. In [22], the authors considered a worst-case scenario in which they assumed that all links are NLOS. Based on this, and the assumption that the magnitude of the NLOS bias is (imperfectly) known, a min-max problem was derived and a robust estimator given in a generalized trust region sub-problem (GTRS) framework was proposed.

This work is inspired by the recently published work in [20], where the authors argued that fusion of RSS and TOA measurements does not necessarily bring benefit by default in comparison with RSS-only and/or TOA-only measurements. Based on a theoretical analysis, they determined a formula to compute the value of a *critical distance*, around which the hybrid measurements should offer gain, whereas for links far below or above this value, TOA-only and RSS-only measurements should dominate, respectively. It is a well known fact that RSS measurements are beneficial for short-ranges, whereas the TOA ones are well suited for long-range links [1]. Hence, the intuition presented in [20] is that if one measurement is much more accurate than the other one, there is little or no advantage in coupling them together, i.e., the more accurate measurement should be employed only. Nevertheless, in practice, the real challenge in this case is to know which of the measurements is more accurate.

Even though the authors in [20] derived a formula to determine the critical distance, this formula is based on accurate knowledge of certain parameters (for instance, noise powers and path loss exponent (PLE)). This assumption is at least optimistic, since it is very unlikely that it stands in practice. Moreover, they considered a ranging problem based on hybrid RSS and TOA quantities, where the main goal was to estimate the distance of each link in line-of-sight (LOS) environments only. In huge contrast to [20], here we consider target localization problem, i.e., our goal is to determine the estimation of target’s location. To do so, both RSS and TOA observations are employed and mixed LOS/NLOS environments are investigated. We exploit the state-of-the-art estimators in [18,19,20,21,22] for the problem of interest and we study their performance in different scenarios and compare it with their counterparts that employ RSS-only and TOA-only measurements. In addition, we derive a novel heuristic approach to select *the best* measurement for each link based on relative difference in RSS and TOA measurements, which we test on the estimator presented in [21]. This heuristic approach does not depend on the knowledge about noise powers, and can match or even better the performance of the best individual option, which highly differs in considered scenarios.

## 2. Problem Formulation

Let us consider a *p*-dimensional (p=2 or 3) sensor network comprising *N* reference sensors with known locations (called anchors) and a sensor whose location we wish to determine (called target). It is assumed that the target emits a signal to anchors, which are suitably equipped to withdraw RSS and TOA information from the received signal.

According to [2,3,13,18,21,23,25,26], RSS and TOA in NLOS conditions can be modeled as

(1a) $$\begin{array}{c} P_{i}=P_{0}-b_{i}-10\gamma \log_{10}\frac{∥x-a_{i}∥}{d_{0}}+n_{i}, \end{array}$$

(1b) $$\begin{array}{c} d_{i}=∥x-a_{i}∥+\beta_{i}+m_{i}, \end{array}$$

respectively, where $P_{0}$ (dBm) is the target’s transmit power, $b_{i}$ (dB) and $\beta_{i}$ (m) are the (positive) NLOS biases ($b_{i}=\beta_{i}=0$ if $i\in L_{l}$, and $b_{i},\beta_{i}>0$ if $i\in L_{n}$, with $L_{l}$, $L_{n}$ representing the set of all LOS and NLOS links, respectively), γ is the path loss exponent, $x,a_{i}$ represent the true target’s and *i*-th anchor’s location (i=1,...,N), respectively, $d_{0}$ is a reference distance ($∥x-a_{i}∥\geq d_{0}$), $n_{i}$ is the log-normal shadowing term (dB) modeled as a zero-mean Gaussian random variable with variance $\sigma_{n_{i}}^{2}$, i.e., $n_{i}∼N\left(0,\sigma_{n_{i}}^{2}\right)$, and $m_{i}$ is the measurement noise (m) modeled as $m_{i}∼N\left(0,\sigma_{m_{i}}^{2}\right)$. Moreover, many authors assume that the magnitudes of the NLOS biases are bounded by a known constant [5,7,17], i.e., $0\leq b_{i}\leq b_{\max}$ and $0\leq \beta_{i}\leq \beta_{\max}$.

If all RSS and TOA measurements are stacked in a single vector, i.e., $P={\left[P_{i}\right]}^{T}$ and $d={\left[d_{i}\right]}^{T}$ ($P,d\in R^{N}$), one can write the joint likelihood function as

(2) $$\begin{array}{c} \Lambda \left(P,d|x,b_{i},\beta_{i}\right)=p\left(P|x,b_{i}\right)\;p\left(d|x,\beta_{i}\right)=\frac{1}{\sqrt{2\pi \sigma_{n_{i}}^{2}\sigma_{m_{i}}^{2}}}\\ \;\exp\;\left\{\;-\frac{{\left(P_{i}-P_{0}+b_{i}+10\gamma \log_{10}\frac{∥x-a_{i}∥}{d_{0}}\right)}^{2}\sigma_{m_{i}}^{2}+{\left(d_{i}-∥x-a_{i}∥-\beta_{i}\right)}^{2}\sigma_{n_{i}}^{2}}{\sigma_{n_{i}}^{2}\sigma_{m_{i}}^{2}}\;\right\}\;, \end{array}$$

with p(•) representing the probability density function (PDF). If the RSS and TOA measurements are taken from independent sources, the above equation is the exact likelihood function [20]. This assumption is not unreasonable, as the authors in [23,27] showed by performing experimental measurements; the observations withdrawn from the same signal are weakly correlated.

If one maximizes the joint PDF of the RSS and TOA observations [10,12,14,28,29,30], the hybrid maximum likelihood (ML) estimator of x, $b_{i}$ and $\beta_{i}$ is derived as

(3) $$\begin{array}{c} \left\{\hat{x},{\hat{b}}_{i},{\hat{\beta }}_{i}\right\}=\underset{x,b_{i},\beta_{i}}{\arg\;\min}\sum_{i=1}^{N}\\ \frac{{\left(P_{i}-P_{0}+b_{i}+10\gamma \log_{10}\frac{∥x-a_{i}∥}{d_{0}}\right)}^{2}\sigma_{m_{i}}^{2}+{\left(d_{i}-∥x-a_{i}∥-\beta_{i}\right)}^{2}\sigma_{n_{i}}^{2}}{\sigma_{n_{i}}^{2}\sigma_{m_{i}}^{2}}. \end{array}$$

The problem in (3) is highly non-convex and does not have a closed-form solution. Furthermore, in practice, it is hard to distinguish between LOS/NLOS links; hence, (3) is also under-determined, since the number of unknowns (2N+p) is greater than the number of observations (2N). Therefore, in order to solve (3), some approximations are required. In the following section, we present various solutions available in the literature that circumvent the non-convexity of (3) by applying different approaches.

## 3. Target Localization Using Integrated RSS and TOA Measurements

In this section, a brief overview of the existing localization algorithms based on combined RSS and TOA measurements are presented first [14,18,20,21,23]. These algorithms serve as the state-of-the-art for the considered problem, and will be used in Section 5 to acquire a set of simulation results for the purpose of our discussion. Then, a heuristic approach to choose the best measurement (e.g., RSS-only, TOA-only or RSS-TOA) for each link, based on relative difference of the estimated distance from the gathered RSS and TOA observations, is proposed.

### 3.1. HWLS Algorithm

The authors in [18] first assumed that they can distinguish between LOS/NLOS links by using a Nakagami-*m* distribution. Nevertheless, note that perfect distinction between LOS/NLOS links is almost impossible in practice. Then, they disregarded the noise and derived the estimated ranges based on the RSS and TOA measurements in (1a) and (1b), i.e.,

(4a) $$\begin{array}{c} {\hat{r}}_{i}^{RSS}=d_{0}10^{\frac{P_{0}-P_{i}-b_{i}}{10\gamma }}, \end{array}$$

(4b) $$\begin{array}{c} {\hat{r}}_{i}^{TOA}=d_{i}-\beta_{i}. \end{array}$$

After squaring (4a) and (4b) and applying simple algebraic manipulations, the following relation was established, written in a matrix notation.

(5) Gθ=h,

where

$$G=\left[\begin{array}{cc} a_{1}^{T} & -\frac{1}{2}\\ \vdots & \vdots \\ a_{N}^{T} & -\frac{1}{2}\\ a_{1}^{T} & -\frac{1}{2}\\ \vdots & \vdots \\ a_{N}^{T} & -\frac{1}{2} \end{array}\right],\;\theta =\left[\begin{array}{c} x\\ Q \end{array}\right],\;h=\left[\begin{array}{c} \frac{1}{2}\left(∥a_{1}{∥}^{2}-{\left({\hat{r}}_{1}^{RSS}\right)}^{\frac{2}{\gamma }}\right)\\ \vdots \\ \frac{1}{2}\left(∥a_{N}{∥}^{2}-{\left({\hat{r}}_{N}^{RSS}\right)}^{\frac{2}{\gamma }}\right)\\ \frac{1}{2}\left(∥a_{1}{∥}^{2}-{\left({\hat{r}}_{1}^{TOA}\right)}^{2}\right)\\ \vdots \\ \frac{1}{2}\left(∥a_{N}{∥}^{2}-{\left({\hat{r}}_{N}^{TOA}\right)}^{2}\right) \end{array}\right],$$

with $Q={∥x∥}^{2}$.

The authors in [18] then considered a noisy environment, and from (5), they obtained a solution to the localization problem by solving the following LS problem:

(6) $$\hat{\theta }=\underset{\theta }{\arg\;\min}{\left(G\theta -h\right)}^{T}W\left(G\theta -h\right)={\left(G^{T}WG\right)}^{-1}G^{T}Wh,$$

where W=SJS is the weight matrix, and $S=\mathrm{diag}\left(\left[\frac{{\left({\hat{r}}_{1}^{RSS}\right)}^{2-\gamma }}{\gamma },\cdots ,\;\frac{{\left({\hat{r}}_{N}^{RSS}\right)}^{2-\gamma }}{\gamma },\;\;\;{\hat{r}}_{1}^{TOA},\cdots ,\;{\hat{r}}_{N}^{TOA}\right]\right)$, $J=\mathrm{diag}\left(\left[\sigma_{n_{1}}^{2},\cdots ,\;\sigma_{n_{N}}^{2},\;\;\;\sigma_{m_{1}}^{2},\cdots ,\;\sigma_{m_{N}}^{2}\right]\right)$.

It is worth mentioning that the authors in [18] assume perfect knowledge of the noise powers, which might not be the case in practice.

### 3.2. NR Algorithm

In [23], the authors considered the problem of range estimation between two sensors using RSS and TOA measurements. By exploiting the hybrid measurements, they found the optimal distance estimation, ${\hat{d}}_{i}^{opt}$, by setting derivative of the ML estimator to zero, i.e., they get ${\hat{d}}_{i}^{opt}$ as the non-zero solution of the following equation:

(7) $${\hat{d}}_{i}^{opt}=B_{1i}+B_{2i}∥x-a_{i}∥+B_{3i}∥x-a_{i}{∥}^{2}+B_{4i}\log∥x-a_{i}∥,\;\;\;i=1,\cdots ,N,$$

where $B_{1i}=\frac{20\gamma {\left(\sigma_{m_{i}}\frac{10}{3}\right)}^{2}}{\ln10}\left(P_{i}-P_{0}+b_{i}\right)$, $B_{2i}=-\frac{20}{3}\sigma_{n_{i}}^{2}\left(d_{i}-\beta_{i}\right)$, $B_{3i}=\frac{200}{9}\sigma_{n_{i}}^{2}$, and $B_{4i}=\frac{200\gamma^{2}{\left(\sigma_{m_{i}}\frac{10}{3}\right)}^{2}}{\ln10}$.

However, the problem in (7) does not have a closed-form solution, and the authors in [23] use the Newton-Raphson mehod instead to get a sub-optimal solution. They first obtain a rough estimate of the distance using only the TOA measurements, ${\hat{r}}_{i}^{TOA}$. Then, by drawing the tangent line to the curve of (7) at the point $\left({\hat{r}}_{i}^{TOA},{\hat{d}}_{i}^{opt}{|}_{∥x-a_{i}∥={\hat{r}}_{i}^{TOA}}\right)$ one obtains a better estimate as the intersection of the tangent and the abscissa, i.e.,

(8) $${\hat{d}}_{i}^{NR}=\frac{B_{1i}+B_{2i}{\hat{r}}_{i}^{TOA}+B_{3i}{\left({\hat{r}}_{i}^{TOA}\right)}^{2}+B_{4i}\log{\hat{r}}_{i}^{TOA}}{B_{2i}+B_{3i}{\hat{r}}_{i}^{TOA}+\frac{B_{4i}}{{\hat{r}}_{i}^{TOA}\ln10}}.$$

The estimation can be further improved by repeating this procedure [23].

Note that perfect knowledge of noise powers and NLOS bias realization is assumed known in [23], which might not be a valid assumption in practice.

### 3.3. JAH Algorithm

Similarly to [23], the authors in [20] study the problem of range estimation between two sensors. To derive a sub-optimal estimator, the authors in [20] start by taking the derivative of the ML estimator and setting it to zero. After manipulating the result, they obtain

(9) $$\log∥x-a_{i}∥=-\frac{\gamma^{2}}{a^{2}}∥x-a_{i}{∥}^{2}+\frac{\gamma^{2}{\hat{r}}_{i}^{TOA}}{a^{2}}∥x-a_{i}∥-\frac{P_{i}-P_{0}+b_{i}}{a},\;\;\;i=1,\cdots ,N,$$

where $\gamma =\frac{\sigma_{n_{i}}}{\sigma_{m_{i}}}$ and $a=\frac{10\gamma }{\ln10}$.

The right-hand side of (9) is then relaxed into an affine function. However, since there are infinite possible choices to do so, the authors in [20] chose *n* “control points”, $\delta_{j}$, j=1,⋯,n, to get a general result, and applied an ordinary least squares approach to minimize the parameters of the affine function.

Finally, by assuming that ${\hat{r}}_{i}^{TOA}<\delta =\frac{\delta_{3}-\delta_{1}\delta_{2}}{\delta_{2}-\delta_{1}^{2}}$, where $\delta_{k}=\frac{1}{n}\sum_{j=1}^{n}\delta_{j}^{k}$, k=1,2,3, they derived the following estimator:

(10) $${\hat{d}}_{i}^{JAH}=\frac{1}{\varphi_{i}}W_{0}\left(\varphi_{i}e^{\psi_{i}}\right),$$

where $\varphi_{i}=\frac{\gamma^{2}}{a^{2}}\left({\hat{r}}_{i}+\delta \right)$, $\psi_{i}=\frac{\gamma^{2}}{a^{2}}\left(\frac{a}{\gamma^{2}}\left(P_{0}-P_{i}-b_{i}\right)+\tilde{\delta }\right)$, with $\tilde{\delta }=\delta_{2}-\delta_{1}\delta$ and $W_{0}\left(•\right)$ denoting the principal branch of the Lambert W-function.

It is worth mentioning that in [20], uniformly spaced points were chosen within a predefined interval. In addition, the authors in [20] generalized their estimator for the case where the condition ${\hat{r}}_{i}^{TOA}<\delta =\frac{\delta_{3}-\delta_{1}\delta_{2}}{\delta_{2}-\delta_{1}^{2}}$ is not met. Furthermore, the authors in [20] assume perfect knowledge of noise powers is available and that all links are LOS, which might not be the case in practice.

Even though NR and JAH algorithms were originally designed for estimating the range between two sensors, their generalization to the localization problem is straightforward after one has the range estimates. According to [24], by using the estimates ${\hat{d}}_{i}^{NR}$ and ${\hat{d}}_{i}^{JAH}$ obtained from [23] and [20] respectively, one can obtain a target location estimate by solving the following problem:

(11) $$\underset{x}{\mathrm{minimize}}\;\sum_{i=1}^{N}\omega_{i}{\left(∥x-a_{i}{∥}^{2}-{\hat{d}}_{i}^{NR/JAH}\right)}^{2},$$

where $\omega_{i}=1-\frac{{\hat{d}}_{i}^{NR/JAH}}{\sum_{i=1}^{N}{\hat{d}}_{i}^{NR/JAH}}$.

The problem in (11) can be rewritten in a vector form as

(12) $$\underset{\theta =[x^{T},∥x{∥}^{2}]}{\mathrm{minimize}}\left\{∥\Omega \left(H\theta -g\right){∥}^{2}:\theta^{T}D\theta +2f^{T}\theta \right\},$$

where Ω=diag(ω), with $\omega =\sqrt{\omega_{i}}$, and

$$H=\left[\begin{array}{c} -2a_{i}^{T},1\\ \vdots \\ -2a_{N}^{T},1 \end{array}\right],\;g=\left[\begin{array}{c} {\left({\hat{d}}_{1}^{NR/JAH}\right)}^{2}-{∥a_{1}∥}^{2}\\ \vdots \\ {\left({\hat{d}}_{N}^{NR/JAH}\right)}^{2}-{∥a_{N}∥}^{2} \end{array}\right],\;D=\left[\begin{array}{cc} I_{p} & 0_{p\times 1}\\ 0_{1\times p} & 0 \end{array}\right],\;f=\left[\begin{array}{c} 0_{p\times 1}\\ -1/2 \end{array}\right].$$

Observe that both the objective function and the constraint in (12) are quadratic. This type of problem is known as GTRS [24], and its *exact* solution is given by

$$\hat{\theta }\left(\lambda \right)={\left(H^{T}\Omega^{T}\Omega H+\lambda D\right)}^{-1}\left(H^{T}\Omega^{T}\Omega g-\lambda f\right),$$

where λ is a unique solution to ϱ(λ)=0, for λ∈I, with $\varrho \left(\lambda \right)=\hat{\theta }{\left(\lambda \right)}^{T}D\hat{\theta }\left(\lambda \right)+2f^{T}\hat{\theta }\left(\lambda \right)$ and the interval $I=\left(-\frac{1}{\lambda_{1}\left(D,H^{T}\Omega^{T}\Omega H\right)},\infty \right)$, where $\lambda_{1}$ is the maximum eigenvalue of a matrix. It is known that ϱ(λ) is strictly decreasing over *I* [24]; thus, the optimal λ can be readily obtained by a bisection procedure.

### 3.4. SR-WLS Algorithm

The authors in [21] tried to mitigate the influence of the NLOS bias in the mean sense, by approximating the *N* NLOS biases by a single (mean) one, which transforms the originally under-determined problem into a determined one (for 2N≥p+2). By using this approximation, from (1a) and (1b) one gets

(13a) $$\begin{array}{c} P_{i}=P_{0}-b-10\gamma \log_{10}\frac{∥x-a_{i}∥}{d_{0}}+n_{i}, \end{array}$$

(13b) $$\begin{array}{c} d_{i}=∥x-a_{i}∥+\beta +m_{i}, \end{array}$$

where *b* and β represent the mean NLOS bias for RSS and TOA measurements, respectively, also called the balancing parameters. Obviously, the price one pays for such an approximation is only partial mitigation of the NLOS bias. However, on the other hand, it allows to keep the balancing parameters as optimization variables to be estimated together with the target location, which leaves somewhat *control* over the problem at hand (e.g., in the two extreme cases: all LOS/NLOS links).

In [21], the authors then rearrange (13a) and apply the first order Taylor series approximation to it. This is followed by squaring the derived equation, together with squaring (13b) and some simple algebraic manipulations to, by introducing weights, finally derive the following WLS problem:

(14) $$\underset{x,b,\beta }{\mathrm{minimize}}\sum_{i=1}^{N}w_{Ri}{\left(\frac{\xi_{i}^{2}{∥x-a_{i}∥}^{2}-\rho^{2}}{2\xi_{i}∥x-a_{i}∥}\right)}^{2}+\sum_{i=1}^{N}w_{Ti}{\left(\frac{∥x-a_{i}{∥}^{2}-{\ddot{d}}_{i}^{2}}{2∥x-a_{i}∥}\right)}^{2},$$

where $\rho =d_{0}10^{\frac{P_{0}-b}{10\gamma }}$, $\xi_{i}=10^{\frac{P_{i}}{10\gamma }}$, $w_{Ri}=1-{\hat{d}}_{i}/\sum_{i=1}^{N}{\hat{d}}_{i}$ and $w_{Ti}=1-{\ddot{d}}_{i}/\sum_{i=1}^{N}{\ddot{d}}_{i}$, where ${\hat{d}}_{i}=d_{0}10^{\frac{P_{0}-P_{i}-b}{10\gamma }}$ represents the ML estimate of the distance from (1a) and ${\ddot{d}}_{i}=d_{i}-\beta$, so that more relevance is given to *nearby* links.

However, since the problem in (14) is non-convex, instead of tackling (14) directly, the authors in [21] substituted it by

(15) $$\underset{x,b,\beta }{\mathrm{minimize}}\sum_{i=1}^{N}w_{Ri}{\left(\frac{\xi_{i}^{2}{∥x-a_{i}∥}^{2}-\rho^{2}}{2\xi_{i}{\hat{d}}_{i}}\right)}^{2}+\sum_{i=1}^{N}w_{Ti}{\left(\frac{∥x-a_{i}{∥}^{2}-{\ddot{d}}_{i}^{2}}{2{\ddot{d}}_{i}}\right)}^{2}.$$

Observe that if *b* and β were known, (15) could be solved *exactly* by a bisection procedure. This motivated the authors in [21] to apply an alternating procedure [6] to estimate x, and *b* and β. The final framework of the problem written as a generalized trust region sub-problem is given below.

(16) $$\begin{array}{c} \underset{\theta =[x^{T}{,∥x∥}^{2}{]}^{T}}{\mathrm{minimize}}\left\{∥W\left(A\theta -q\right){∥}^{2}:\theta^{T}D\theta +2F^{T}\theta =0\right\}, \end{array}$$

where $W=\mathrm{diag}(\left[{\tilde{w}}_{1}^{T},{\tilde{w}}_{2}^{T}\right])$, ${\tilde{w}}_{1}={\left[{\tilde{w}}_{1i}\right]}^{T}$, ${\tilde{w}}_{1i}=\frac{\sqrt{w_{Ri}}}{2\xi_{i}{\overset{˘}{d}}_{i}}$, ${\tilde{w}}_{2}={\left[{\tilde{w}}_{2i}\right]}^{T}$, ${\tilde{w}}_{2i}=\frac{\sqrt{w_{Ti}}}{2{\tilde{d}}_{i}}$,

$$A\;=\;\left[\begin{array}{cc} \vdots & \vdots \\ 2\xi_{i}^{2}a_{i}^{T} & -\xi_{i}^{2}\\ \vdots & \vdots \\ 2a_{i}^{T} & -1\\ \vdots & \vdots \end{array}\right]\;,\;q\;=\;\left[\begin{array}{c} \vdots \\ \xi_{i}^{2}{∥a_{i}∥}^{2}-{\hat{\rho }}^{2}\\ \vdots \\ ∥a_{i}{∥}^{2}-{\tilde{d}}_{i}^{2}\\ \vdots \end{array}\right]\;,$$

After solving the problem in (16) for a fixed *b* and β and obtaining an estimate of the target’s location, $\hat{x}$, the authors in [21] updated their estimates as follows.

$$\hat{b}=\frac{\sum_{i=1}^{N}\left(P_{0}-P_{i}-10\gamma \log_{10}\frac{∥\hat{x}-a_{i}∥}{d_{0}}\right)}{N},$$

$$\hat{\beta }=\frac{\sum_{i=1}^{N}\left(d_{i}-∥\hat{x}-a_{i}∥\right)}{N}.$$

**Figure 1:**
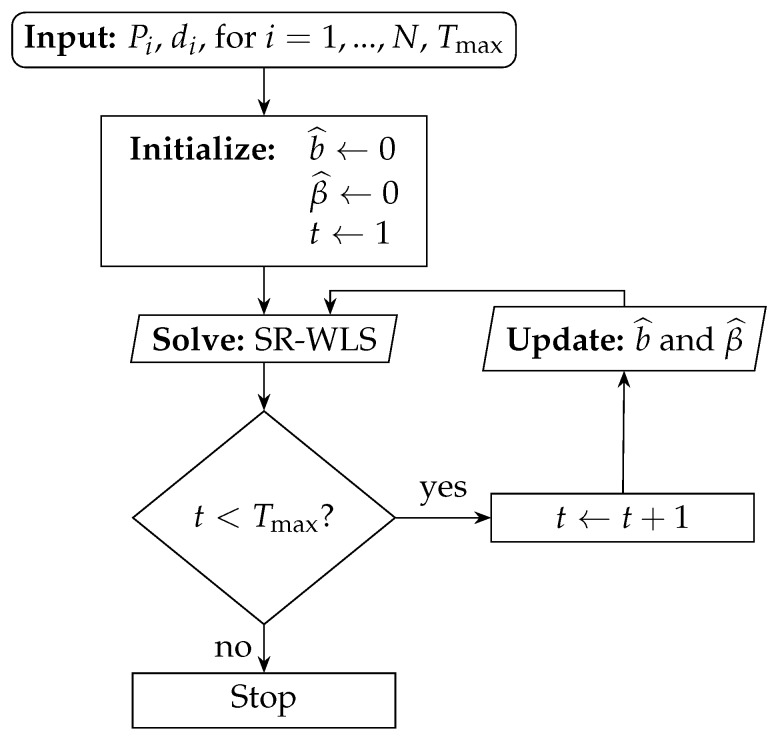
Flow chart diagram of the SR-WLS algorithm in [21].

The alternating procedure is given in a flow chart, presented in Figure 1. For more details, the reader is referred to [21]. As it can be seen from Figure 1, SR-WLS is composed of two main phases: solving the localization problem and updating the NLOS bias estimates. In the first iteration, all links are treated as LOS, i.e., $\hat{b}=\hat{\beta }=0$ is set. With the use of these estimates, the localization problem is solved to acquire an estimation of the target location. Then, by exploiting this estimate, an update of the NLOS bias estimates is performed, and the localization problem is solved again by employing the updated estimates. This alternating procedure is executed $T_{\max}$ times [21].

### 3.5. R-GTRS Algorithm

Unlike [21], where the authors first treated all links as LOS to later apply an alternating optimization approach and improve the location estimate and the mean NLOS bias estimate in an iterative fashion, the authors in [14] took a different (single-step) approach. By treating all links as NLOS and NLOS biases as nuisance parameters whose upper bound on the magnitude is assumed (imperfectly) known, they mitigated their negative influence by resorting to a robust, worst-case scenario, approach.

First, $\frac{b_{\max}}{2}$ was added to both sides of (1a) and $\beta_{\max}/2$ was subtracted from both sides of (1b).

(17a) $$\begin{array}{c} {\tilde{P}}_{i}=P_{0}-{\tilde{b}}_{i}-10\gamma \log_{10}\frac{∥x-a_{i}∥}{d_{0}}+n_{i}, \end{array}$$

(17b) $$\begin{array}{c} {\tilde{d}}_{i}=∥x-a_{i}∥+{\tilde{\beta }}_{i}+m_{i}, \end{array}$$

with ${\tilde{P}}_{i}=P_{i}+b_{\max}/2$, ${\tilde{b}}_{i}=b_{i}-b_{\max}/2$, ${\tilde{d}}_{i}=d_{i}-\frac{\beta_{\max}}{2}$ and ${\tilde{\beta }}_{i}=\beta_{i}-\frac{\beta_{\max}}{2}$.

After doing some simple manipulations with (17a), the first order Taylor series approximation for small noise was applied to this equation. The so derived equation was then squared, together with (17b). After disregarding the second-order noise terms and applying a WLS criterion, the following min-max problem was obtained:

(18a) $$\begin{array}{c} \underset{x}{\mathrm{minimize}}\;\;\underset{\rho_{i}}{\mathrm{maximize}}\sum_{i=1}^{N}{\left(\frac{\nu_{i}^{2}{∥x-a_{i}∥}^{2}-\rho_{i}^{2}}{2\nu_{i}∥x-a_{i}∥}\right)}^{2}, \end{array}$$

(18b) $$\begin{array}{c} \underset{x}{\mathrm{minimize}}\;\;\underset{{\tilde{\beta }}_{i}}{\mathrm{maximize}}\sum_{i=1}^{N}{\left(\frac{{\left({\tilde{d}}_{i}-{\tilde{\beta }}_{i}\right)}^{2}-{∥x-a_{i}∥}^{2}}{2∥x-a_{i}∥}\right)}^{2}, \end{array}$$

which can be written as

(19a) $$\begin{array}{c} \underset{x}{\mathrm{minimize}}\;\;\;\underset{\rho_{i}}{\mathrm{maximize}}\sum_{i=1}^{N}f^{2}\left(\rho_{i}\right),\;\;\;\mathrm{where}\;\;\;f\left(\rho_{i}\right)=\frac{|\nu_{i}^{2}{∥x-a_{i}∥}^{2}-\rho_{i}^{2}|}{2\nu_{i}∥x-a_{i}∥}, \end{array}$$

(19b) $$\begin{array}{c} \underset{x}{\mathrm{minimize}}\;\;\;\underset{{\tilde{\beta }}_{i}}{\mathrm{maximize}}\sum_{i=1}^{N}f^{2}\left({\tilde{\beta }}_{i}\right),\;\;\;\mathrm{where}\;\;\;f\left({\tilde{\beta }}_{i}\right)=\frac{|{\left({\tilde{d}}_{i}-{\tilde{\beta }}_{i}\right)}^{2}-{∥x-a_{i}∥}^{2}|}{2∥x-a_{i}∥}. \end{array}$$

By noticing that

(20) $$|{\tilde{b}}_{i}\left|=\right|b_{i}-\frac{b_{\max}}{2}|\leq \frac{b_{\max}}{2},\;\;\;|{\tilde{\beta }}_{i}\left|=\right|\beta_{i}-\frac{\beta_{\max}}{2}|\leq \frac{\beta_{\max}}{2},$$

and that

(21a) $$\begin{array}{c} \underset{\rho_{i}}{\mathrm{maximize}}\sum_{i=1}^{N}f^{2}\left(\rho_{i}\right)=\sum_{i=1}^{N}{\left[\underset{\rho_{i}}{\mathrm{maximize}}f\left(\rho_{i}\right)\right]}^{2}, \end{array}$$

(21b) $$\begin{array}{c} \underset{{\tilde{\beta }}_{i}}{\mathrm{maximize}}\sum_{i=1}^{N}f^{2}\left({\tilde{\beta }}_{i}\right)=\sum_{i=1}^{N}{\left[\underset{{\tilde{\beta }}_{i}}{\mathrm{maximize}}f\left({\tilde{\beta }}_{i}\right)\right]}^{2}, \end{array}$$

the authors in [14] first solve the maximization problems under certain conditions, where they got two possible solutions. By applying these two solutions, and joining the RSS and TOA branches together into a single estimator, the following robust estimation problem was derived:

(22) $$\begin{array}{c} \underset{x}{\mathrm{minimize}}\sum_{i=1}^{N}{\left(\frac{\nu_{i}^{2}{∥x-a_{i}∥}^{2}-\mu }{2\nu_{i}∥x-a_{i}∥}\right)}^{2}+\sum_{i=1}^{N}{\left(\frac{\nu_{i}^{2}{∥x-a_{i}∥}^{2}-\eta }{2\nu_{i}∥x-a_{i}∥}\right)}^{2}\\ +\sum_{i=1}^{N}{\left(\frac{d_{i}^{2}-{∥x-a_{i}∥}^{2}}{2∥x-a_{i}∥}\right)}^{2}+\sum_{i=1}^{N}{\left(\frac{{\left({\tilde{d}}_{i}-\frac{\beta_{\max}}{2}\right)}^{2}-{∥x-a_{i}∥}^{2}}{2∥x-a_{i}∥}\right)}^{2}. \end{array}$$

Since both RSS and TOA short-distance links are trusted more than the remote ones, due to their multiplicative and additive factors [1], the authors in [14] enhanced the localization accuracy, by introducing weights in (22), defined as $w={\left[{\hat{w}}_{i},{\tilde{w}}_{i}\right]}^{T}$, where ${\hat{w}}_{i}=1-{\hat{d}}_{i}/\sum_{i=1}^{N}{\hat{d}}_{i}$, ${\tilde{w}}_{i}=1-{\tilde{d}}_{i}/\sum_{i=1}^{N}{\tilde{d}}_{i}$, with ${\hat{d}}_{i}=d_{0}10^{\frac{P_{0}-P_{i}-b_{\max}/2}{10\gamma }}$ being a *mean* ML estimate of the distance from (1a). In addition, because (22) is highly non-convex, it was not tackled directly, but it was rather substituted by

(23) $$\begin{array}{c} \underset{x}{\mathrm{minimize}}\sum_{i=1}^{N}{\hat{w}}_{i}{\left(\frac{\nu_{i}^{2}{∥x-a_{i}∥}^{2}-\mu }{2\nu_{i}{\hat{d}}_{i}}\right)}^{2}+\sum_{i=1}^{N}{\hat{w}}_{i}{\left(\frac{\nu_{i}^{2}{∥x-a_{i}∥}^{2}-\eta }{2\nu_{i}{\hat{d}}_{i}}\right)}^{2}\\ +\sum_{i=1}^{N}{\tilde{w}}_{i}{\left(\frac{d_{i}^{2}-{∥x-a_{i}∥}^{2}}{2{\tilde{d}}_{i}}\right)}^{2}+\sum_{i=1}^{N}{\tilde{w}}_{i}{\left(\frac{{\left({\tilde{d}}_{i}-\frac{\beta_{\max}}{2}\right)}^{2}-{∥x-a_{i}∥}^{2}}{2{\tilde{d}}_{i}}\right)}^{2}. \end{array}$$

Then, by expanding the squared norm terms in the numerators of (23), the proposed joint hybrid localization algorithm can be written as

(24) $$\underset{\theta =[x^{T}{,∥x∥}^{2}{]}^{T}}{\mathrm{minimize}}\left\{∥\tilde{W}\left(\tilde{A}\theta -\tilde{q}\right){∥}^{2}\;:\;\;\;\theta^{T}D\theta +2f^{T}\theta =0\right\},$$

$\tilde{W}=\mathrm{diag}\left(\left[w_{1}^{T},w_{2}^{T}\right]\right)$, $w_{1}={\left[w_{1i},w_{1i}\right]}^{T}$, $w_{1i}=\frac{\sqrt{{\hat{w}}_{i}}}{2\nu_{i}{\hat{d}}_{i}}$ and $w_{2}={\left[w_{2i},w_{2i}\right]}^{T}$, $w_{2i}=\frac{\sqrt{{\tilde{w}}_{i}}}{2{\tilde{d}}_{i}}$ for i=1,...,N,

$$\tilde{A}=\left[\begin{array}{cc} \vdots & \vdots \\ -2\nu_{i}^{2}a_{i}^{T} & \nu_{i}^{2}\\ \vdots & \vdots \\ -2\nu_{i}^{2}a_{i}^{T} & \nu_{i}^{2}\\ \vdots & \vdots \\ 2a_{i}^{T} & -1\\ \vdots & \vdots \\ 2a_{i}^{T} & -1\\ \vdots & \vdots \end{array}\right],\;\tilde{q}=\left[\begin{array}{c} \vdots \\ \mu -\nu_{i}^{2}{∥a_{i}∥}^{2}\\ \vdots \\ \eta -\nu_{i}^{2}{∥a_{i}∥}^{2}\\ \vdots \\ ∥a_{i}{∥}^{2}-d_{i}^{2}\\ \vdots \\ ∥a_{i}{∥}^{2}-{\left({\tilde{d}}_{i}-\frac{\beta_{\max}}{2}\right)}^{2}\\ \vdots \end{array}\right].$$

### 3.6. A Heuristic Approach for the Best Measurement Selection

The authors in [20] introduced a term *critical distance*, which is the distance between two sensors around which the performance of estimators based on hybrid RSS-TOA measurements should outperform both estimators based on RSS-only and TOA-only measurements. According to [20], the formula for calculating the critical distance is given as

(25) $$D=\frac{\sigma_{m_{i}}}{\sqrt{e^{\frac{\sigma_{n_{i}}^{2}}{a}}-1}}.$$

However, the formula in (25) does not depend on the size of the area where the sensors are deployed, and it assumes perfect knowledge of the noise powers. The latter assumption might not stand in practice; thus, a different approach is considered here.

From (1a) and (1b) respectively, the distance that best estimates $∥x-a_{i}∥$ in the mean ML sense is

(26a) $$\begin{array}{c} {\hat{d}}_{i}^{RSS}=10^{\frac{P_{0}-P_{i}-b_{\max}/2}{10\gamma }}, \end{array}$$

(26b) $$\begin{array}{c} {\hat{d}}_{i}^{TOA}=d_{i}-\beta_{\max}/2. \end{array}$$

By taking advantage of the estimates in (26a) and (26b), we can calculate the normalized relative difference between them as

(27) $$\epsilon =\frac{|{\hat{d}}_{i}^{RSS}-{\hat{d}}_{i}^{TOA}|}{\max\{{\hat{d}}_{i}^{RSS},{\hat{d}}_{i}^{TOA}\}}.$$

As it is well known, RSS measurements are most valuable for short inter-sensor distances, while TOA ones bring advantage for long inter-sensor distances [1]. In order to exploit this fact, we make use of (27) to derive one of the following three choices:

- $\epsilon <\epsilon_{\min}$: use TOA-only measurements;
- $\epsilon_{\max}\leq \epsilon \leq \epsilon_{\max}$: use hybrid RSS-TOA measurements;
- $\epsilon >\epsilon_{\max}$: use RSS-only measurements.

The values chosen for $\epsilon_{\min}$ and $\epsilon_{\max}$ in the above approach are entirely based on heuristics, but they make sense in what we consider to be a short and a long inter-sensor distance (as it will be seen in Section 5). Furthermore, our approach does not depend on the knowledge about the noise powers and it implicitly incorporates the size of the area, i.e., the length of links.

## 4. Complexity Analysis

Given *K* as the maximum number of steps in the bisection procedure, Table 1 summarizes the computational complexities of the considered algorithms in this work. It can be seen from Table 1 that all estimators have linear computational complexity in *N*. Nevertheless, it is worth mentioning that JAH requires computing the principal branch of the Lambert W-function [20] and that SR-WLS is executed iteratively [21]; hence the execution time of these estimators is somewhat higher than the remaining ones.

## 5. Simulation Results

In this section, a set of simulation results are presented with the objective to analyze the performance of the considered algorithms and compare them with the performance of their counterparts that make use of RSS-only and TOA-only observations. Note that HWLS in [18], NR in [23] and JAH [20] were all implemented here with perfect knowledge of noise powers and perfect knowledge of $b_{i}$ and $\beta_{i}$, which does not hold in practice. Hence, the results presented herein can be seen as lower bounds for these estimators, rather than their true achievable performance. All presented results were acquired by using MATLAB. Two main scenarios are considered: (1) random deployment of all sensors inside a quadratic region of length *B* in each Monte Carlo, $M_{c}$, run, and (2) fixed placement of anchors according to Table 2 and random deployment of the target within a quadratic region of length $B_{t}$. The second scenario is adopted here with the purpose of guaranteeing a steady distance between the target and anchors. Note that the first *N* anchors were always employed for this scenario, in concordance with Table 2. RSS and TOA measurements were generated according to (1a) and (1b). Unless stated otherwise, fixed simulation parameters are summarized in Table 3. For the ease of expression, $\sigma_{i}$ (dB, m) and ${\mathrm{bias}}_{i}$ (dB, m) are used to denote the noise powers and the NLOS biases of both the RSS and TOA measurements, respectively. Moreover, the NLOS biases were drawn randomly from an exponential distribution whose rate parameter is drawn from a uniform distribution on the interval $\left[0,{\mathrm{bias}}_{\max}\right]$ (dB, m), i.e., ${\mathrm{bias}}_{i}∼\mathrm{Exp}\left(U\left[0,{\mathrm{bias}}_{\max}\right]\right)$, i=1,...,N, in each $M_{c}$ run. The main performance metric is the root mean squared error (RMSE), $\mathrm{RMSE}=\sqrt{\sum_{i=1}^{M_{c}}\frac{∥x_{i}-{\hat{x}}_{i}∥}{M_{c}}}$, where ${\hat{x}}_{i}$ denotes the estimate of the true target location, $x_{i}$, in the *i*-th $M_{c}$ run.

**Figure 2:**
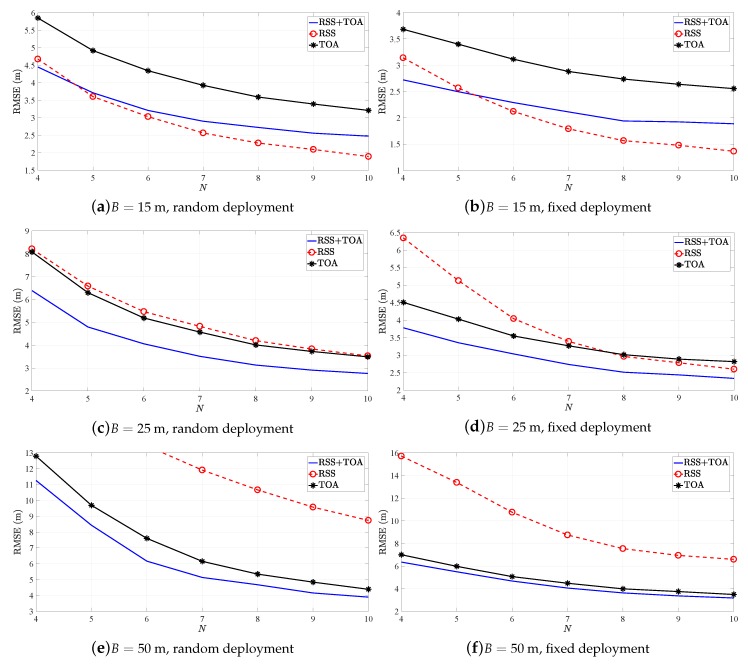
Root mean squared error (RMSE) versus *N* comparison for R-GTRS.

Figure 2 illustrates the RMSE (m) versus *N* performance of R-GTRS estimator in [22] for different *B* in the two considered scenarios. The figure shows that the counterpart of the estimator employing RSS-only measurements is the best option for low *B* in general. However, when *B* is increased this counterpart worsens significantly, as expected, and the hybrid version of the estimator becomes the best option. Actually, one can see that measurement integration is the best choice when *N* is low for all *B*. This is not surprising, since it benefits from the double information gathered by each anchor, whereas the *traditional* counterparts do not have that luxury, and have a limited amount of information. When *B* is set to its highest considered value, it was expected that the TOA-only counterpart outperforms the other options; however, this is not quite the case, especially in the first considered scenario. This can be explained to some extent by the fact that, in the first scenario, random deployment of all sensors is considered and there were no guaranties that the actual inter-sensor distance was large. Nevertheless, this can not be said in the second scenario, where the hybrid estimator and its TOA-only counterpart exhibit practically the same performance.

**Figure 3:**
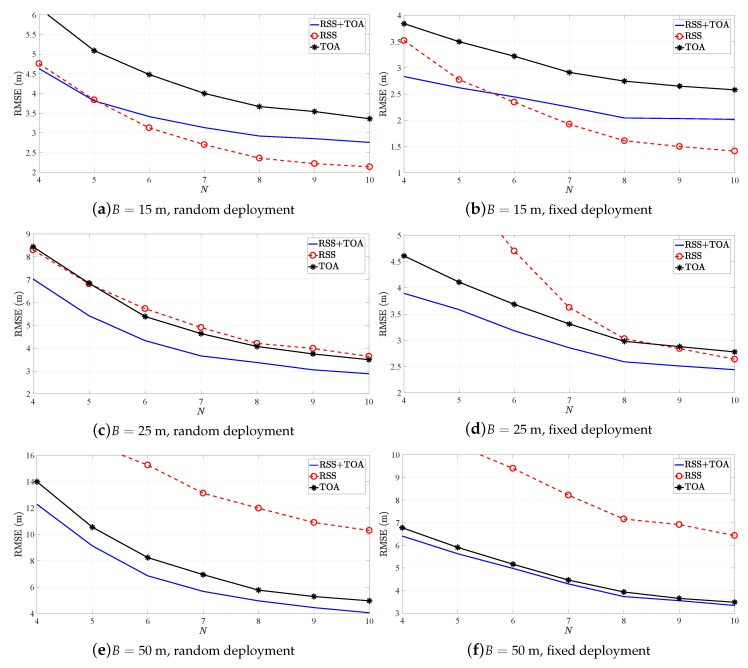
RMSE versus *N* comparison for SR-WLS.

Figure 3 illustrates the RMSE (m) versus *N* performance of SR-WLS estimator in [21] for different *B* in the two considered scenarios. Principally, very similar conclusions to those of Figure 2 can be made from Figure 3: the best option for low *B* is the RSS-only counterpart in general, whereas the hybrid estimator dominates over the remaining ones as *B* is increased. Once again, the TOA-only counterpart practically matches the performance of the hybrid estimator in the second scenario for the highest considered value of *B*.

**Figure 4:**
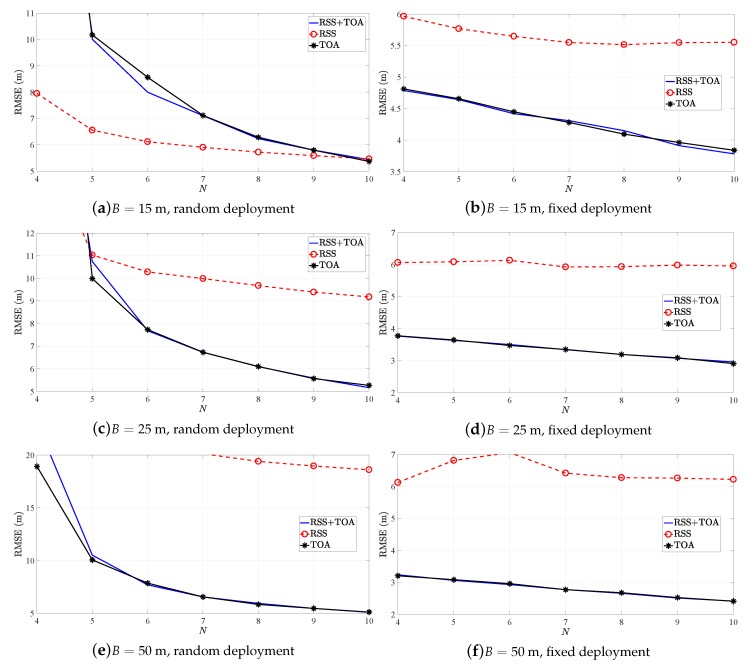
RMSE versus *N* comparison for HWLS.

Figure 4 illustrates the RMSE (m) versus *N* performance of HWLS estimator in [18] for different *B* in the two considered scenarios. For the first scenario, the figure corroborates earlier conclusions, but leaves the RSS-only counterpart as the worst option in the second scenario for all considered *B*. This result was not anticipated, and no explanation for this behavior is provided here.

**Figure 5:**
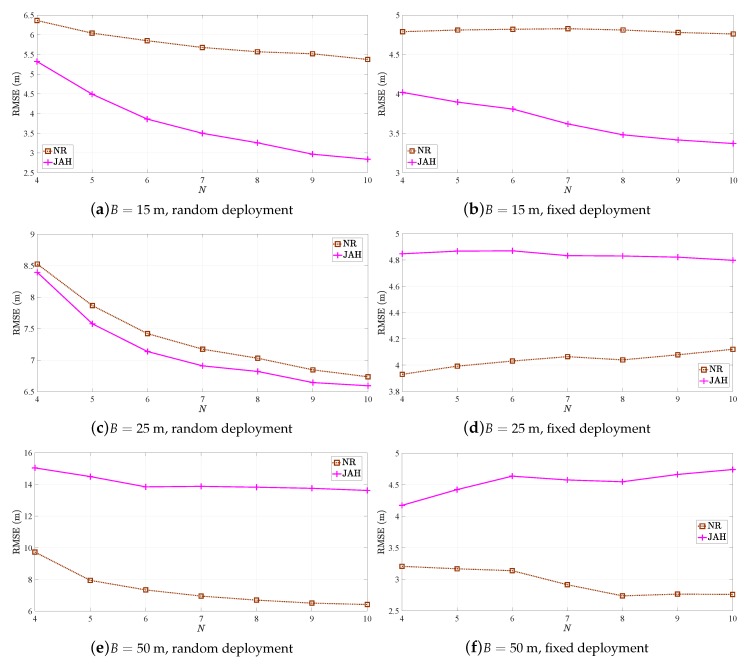
RMSE versus *N* comparison for NR and JAH.

Figure 5 illustrates the RMSE (m) versus *N* performance of NR and JAH estimators in [20,23] respectively, for different *B* in the two considered scenarios. Note that the two estimators utilize both RSS and TOA measurements to obtain the range between the target and anchors, and that their decomposition into RSS-only and TOA-only counterparts is not straightforward. Therefore, only the hybrid version of the algorithms is considered here. Figure 5 exhibits that JAH is favorable in scenarios with low *B*, whereas NR is a better option than JAH when *B* is increased.

**Figure 6:**
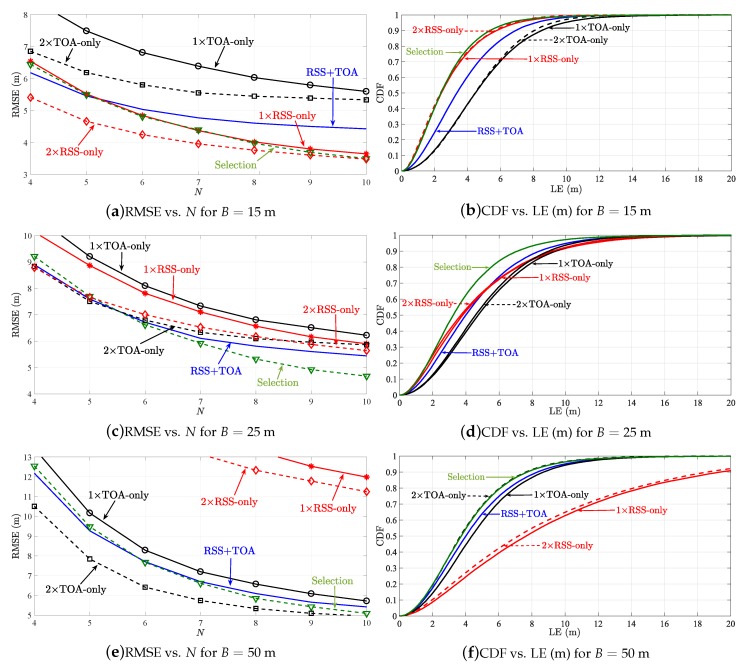
Simulation results for SR-WLS, when ${\mathrm{bias}}_{\max}=6$ (dB, m), ${\mathrm{bias}}_{i}∼U\left[0,{\mathrm{bias}}_{\max}\right]$ (dB, m).

Moreover, Figure 6 illustrates the RMSE (m) versus *N* comparison of SR-WLS estimator in [21] in the first considered scenario when NLOS biases are randomly chosen from a uniform distribution. It also illustrates a comparison of cumulative distribution function (CDF) versus localization error (LE), defined as $∥{\hat{x}}_{i}-x_{i}∥$ (m) in the same scenario. The figure presents the results of the estimator when it uses the proposed heuristic approach, denoted as “Selection”, as well as the results of the estimator utilizing double RSS-only and double TOA-only measurements. Although the comparison in terms of quantity of the acquired information is fair, it is important to note that, in order to acquire the double measurements, SR-WLS_RSS_ and SR-WLS_TOA_ require two signal transmissions. This might affect the sensors’ battery lives in the long term, as well as the utilization efficiency of the radio spectrum (doubling the measurement time and increasing the risk of message collisions). On the other hand, the hybrid algorithms require a single transmission to acquire two measurements (RSS and TOA), at a cost of a somewhat increased complexity of the sensors in terms of hardware. Nevertheless, recent advances in micro electro-mechanical systems allow practically all modern devices to measure these two quantities [1,10].

From Figure 6 one can see that our procedure for choosing *the best* alternative for each link works well, since such estimator matches or even betters the performance of the previously best option in all considered scenarios. This estimator consistently shows the best performance, while a tendency of the estimators using RSS-only and TOA-only observations to worsen and improve as *B* increases is noticed, respectively. Please note that in this work, NLOS environments were considered, whereas the authors in [20] considered that all links were LOS. Although our general findings are in line with the ones presented there, the difference in the considered scenarios might explain why they do not have a perfect alignment in the case where *B* is large.

**Figure 7:**
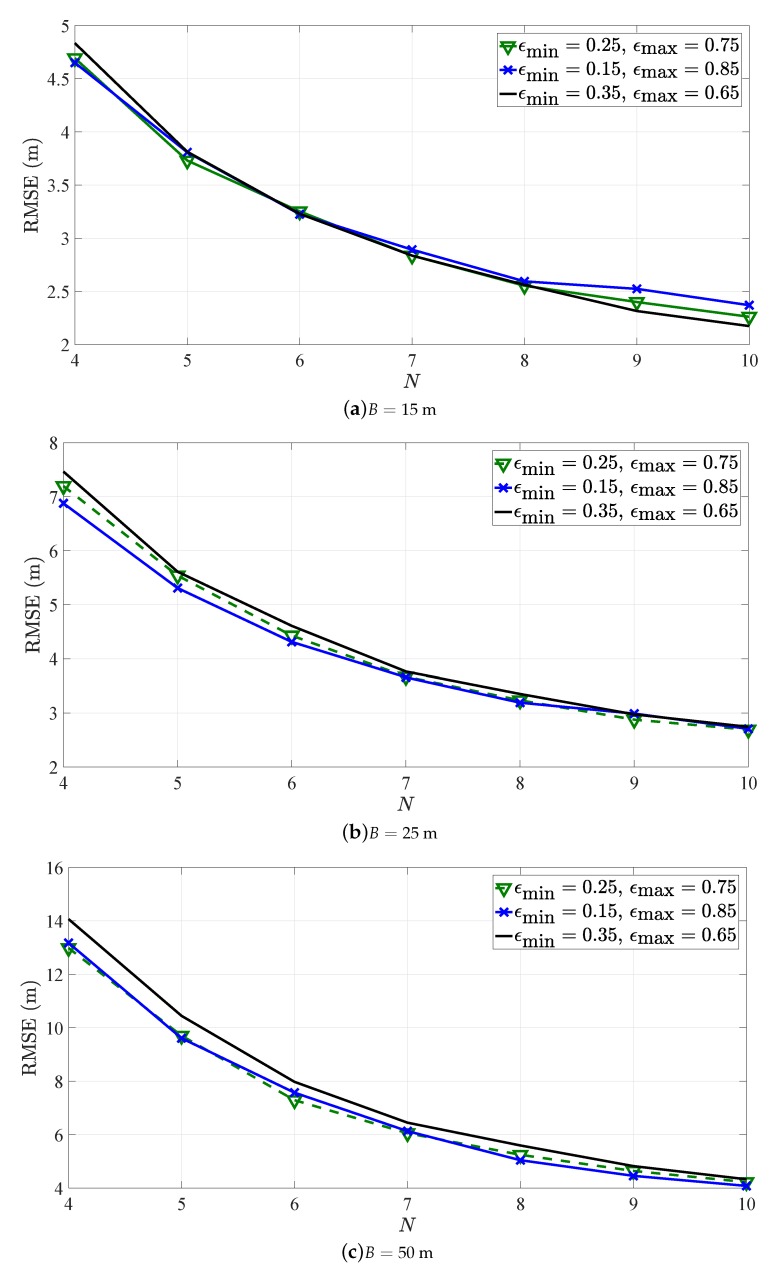
RMSE versus *N* comparison for SR-WLS for different limits for ϵ in the first considered scenario.

In order to check the robustness of the proposed heuristic approach to different values of $\epsilon_{\min}$ and $\epsilon_{\max}$, the RMSE versus *N* for the heuristic approach applied to SR-WLS algorithm in the first considered scenario for different set of values of these parameters is presented in Figure 7. Note that all three sets of values chosen as the limits for ϵ are reasonable in the sense that they capture what is considered to be short/long inter-sensor distance. It can be seen from Figure 7 that there is only marginal difference in the performance for different settings of the parameters of interest, indicating that the proposed heuristic approach is robust to them.

**Figure 8:**
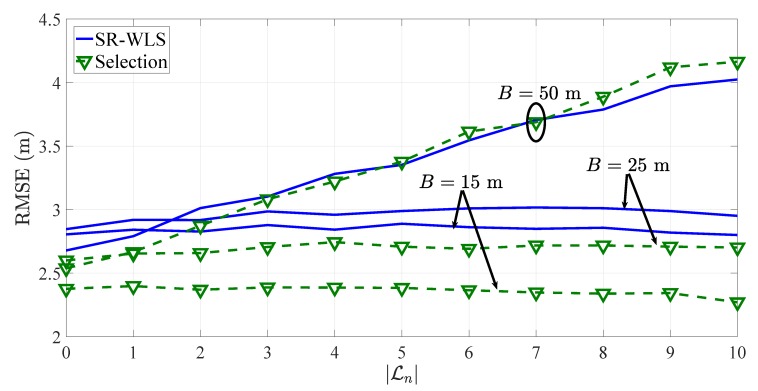
RMSE versus $|L_{n}|$ comparison in the first considered scenario, when N=10, $\gamma_{i}∼U\left[2.7,3.3\right]$, $\sigma_{i}=6$ (dB, m), and ${\mathrm{bias}}_{\max}=6$ (dB, m).

In practice, it is likely to have a mixture of LOS/NLOS links; hence, it is of interest to study the performance of an algorithm against different values of $|L_{n}|$. To do so, the RMSE versus $|L_{n}|$ performance comparison of the proposed heuristic approach applied to SR-WLS in the first considered scenario when N=10 is presented in Figure 8. Moreover, to account for realistic measurement model mismatch and the robustness of the proposed heuristic approach to imperfect knowledge of the PLE, in Figure 8 the PLE is chosen randomly for each link, i.e., it is chosen from a uniform distribution from the interval [2.7,3.3], $\gamma_{i}∼U\left[2.7,3.3\right]$. Figure 8 shows that the proposed heuristic approach is robust to the number of LOS/NLOS links in general. However, in the case where B=50 m, one can notice a clear tendency of its performance to degrade as the number of NLOS links is increased. This result is interesting and not foreseen. Although there is no evidence, it is most likely caused by the poor quality of long inter-sensor RSS measurements, which in NLOS environments suffer additional deterioration. Nevertheless, the result shows that the proposed heuristic approach is not perfect and has its vulnerabilities; hence, further improvements are welcomed in order to reach a more robust solution to the problem.

## 6. Conclusions and Future Work

In this work, the problem of target localization in adverse NLOS environments using integrated RSS and TOA measurements was addressed. It was shown that the considered estimators do not profit from the hybrid measurements by default, and that there is a *gray* area that needs to be taken into consideration, which depends on inter-sensor distances. Moreover, a simple heuristic approach was presented in order to choose *the best* measurement for each link, which is then used for performing the localization. It was shown that such an estimator can match or even better the performance of the estimator using one fixed measurement option. Furthermore, the simulation results indicated that the employed TOA model suffers slightly at relatively short inter-sensor distances, but the employed RSS model cannot seem to handle long ones. This does not come as a surprise, but it is a confirmation that the employed RSS model is over-simplistic for long ranges. Finally, note that all estimators considered here are linear ones. Perhaps employing different tools (e.g., convex optimization techniques or multidimensional scaling) which might require higher computational complexity, but could combat better with the negative NLOS bias effect, and thus result in higher localization accuracy might also be worthy of exploring.

Although our general findings are somewhat similar with the ones presented in [20] for the case of LOS links, it becomes obvious that more work is required when the considered environment contains NLOS links. The negative influence of NLOS biases additionally complicate the problem at hand, since they can have a huge impact when estimating the distance of a link. Moreover, even though our procedure implicitly incorporates inter-sensor distance and does not depend on the knowledge of the noise powers (unlike the existing theoretical formula for calculating the critical distance) there are still many open questions to be considered in order to optimize the mechanism for opting between the available measurements and possibly integrating them in a different (*better*) fashion. These are all open questions that deserve more attention and are left for future work. Likewise, simultaneous localization of multiple targets able to cooperate with each other is of interest for future work. Finally, employing Bayesian theory and filters for real-time tracking of a moving target might be an interesting direction for further research.

## Figures and Tables

**Table 1:** Summary of the Considered Algorithms.

Algorithm	Complexity
HWLS in [18]	$O\left(N\right)$
NR in [23]	$O\left(KN\right)$
JAH in [20]	$O\left(KN\right)$
SR-WLS in [21]	$T_{\max}\times O\left(KN\right)$
R-GTRS in [22]	$O\left(KN\right)$

**Table 2:** Fixed anchors locations (m) in the second considered scenario.

*i*	1	2	3	4	5	6	7	8	9	10
$a_{i}$	$\left[\begin{array}{c} 0\\ 0 \end{array}\right]$	$\left[\begin{array}{c} B\\ 0 \end{array}\right]$	$\left[\begin{array}{c} B\\ B \end{array}\right]$	$\left[\begin{array}{c} 0\\ B \end{array}\right]$	$\left[\begin{array}{c} B/2\\ 0 \end{array}\right]$	$\left[\begin{array}{c} B\\ B/2 \end{array}\right]$	$\left[\begin{array}{c} B/2\\ B \end{array}\right]$	$\left[\begin{array}{c} 0\\ B/2 \end{array}\right]$		
$a_{i}$	$\left[\begin{array}{c} 0\\ 0 \end{array}\right]$	$\left[\begin{array}{c} B\\ 0 \end{array}\right]$	$\left[\begin{array}{c} B\\ B \end{array}\right]$	$\left[\begin{array}{c} 0\\ B \end{array}\right]$	$\left[\begin{array}{c} B/3\\ 0 \end{array}\right]$	$\left[\begin{array}{c} B\\ B/2 \end{array}\right]$	$\left[\begin{array}{c} B/2\\ B \end{array}\right]$	$\left[\begin{array}{c} 0\\ B/2 \end{array}\right]$	$\left[\begin{array}{c} 2B/3\\ 0 \end{array}\right]$	
$a_{i}$	$\left[\begin{array}{c} 0\\ 0 \end{array}\right]$	$\left[\begin{array}{c} B\\ 0 \end{array}\right]$	$\left[\begin{array}{c} B\\ B \end{array}\right]$	$\left[\begin{array}{c} 0\\ B \end{array}\right]$	$\left[\begin{array}{c} B/3\\ 0 \end{array}\right]$	$\left[\begin{array}{c} B\\ B/3 \end{array}\right]$	$\left[\begin{array}{c} B/2\\ B \end{array}\right]$	$\left[\begin{array}{c} 0\\ B/2 \end{array}\right]$	$\left[\begin{array}{c} 2B/3\\ 0 \end{array}\right]$	$\left[\begin{array}{c} B\\ 2B/3 \end{array}\right]$

**Table 3:** Summary of the Fixed Simulation Parameters.

Label	Description	Value
$P_{0}$	Reference power	20 (dBm)
γ	Path loss exponent	3
$d_{0}$	Reference distance	1 (m)
${\mathrm{bias}}_{\max}$	Magnitude of NLOS bias	B/5 (dB, m)
$\sigma_{i}$	Noise power	3 (dB, m)
$B_{t}$	Area border for targets in the second scenario	15 m
$|L_{n}|$	Number of NLOS links	*N*
$T_{\max}$	Max number of iteration for SR-WLS	2
$\epsilon_{\min}$	Lower limit for ϵ	0.25
$\epsilon_{\max}$	Upper limit for ϵ	0.75
$M_{c}$	Number of Monte Carlo runs	50,000
